# When supply does not meet demand-ER stress and plant programmed cell death

**DOI:** 10.3389/fpls.2014.00211

**Published:** 2014-06-04

**Authors:** Brett Williams, Jeanmarie Verchot, Martin B. Dickman

**Affiliations:** ^1^Centre for Tropical Crops and Biocommodities, Queensland University of TechnologyBrisbane, QLD, Australia; ^2^Department of Entomology and Plant Pathology, Oklahoma State UniversityStillwater, OK, USA; ^3^Department of Plant Pathology and Microbiology, Institute for Plant Genomics and Biotechnology, Texas A&M UniversityCollege Station, TX, USA

**Keywords:** unfolded protein response, ER, autophagy, stress response and stress tolerance, PCD

## Abstract

The endoplasmic reticulum (ER) is the central organelle in the eukaryotic secretory pathway. The ER functions in protein synthesis and maturation and is crucial for proper maintenance of cellular homeostasis and adaptation to adverse environments. Acting as a cellular sentinel, the ER is exquisitely sensitive to changing environments principally via the ER quality control machinery. When perturbed, ER-stress triggers a tightly regulated and highly conserved, signal transduction pathway known as the unfolded protein response (UPR) that prevents the dangerous accumulation of unfolded/misfolded proteins. In situations where excessive UPR activity surpasses threshold levels, cells deteriorate and eventually trigger programmed cell death (PCD) as a way for the organism to cope with dysfunctional or toxic signals. The programmed cell death that results from excessive ER stress in mammalian systems contributes to several important diseases including hypoxia, neurodegeneration, and diabetes. Importantly, hallmark features and markers of cell death that are associated with ER stress in mammals are also found in plants. In particular, there is a common, conserved set of chaperones that modulate ER cell death signaling. Here we review the elements of plant cell death responses to ER stress and note that an increasing number of plant-pathogen interactions are being identified in which the host ER is targeted by plant pathogens to establish compatibility.

## INTRODUCTION

The endoplasmic reticulum (ER) contains the necessary machinery to ensure quality protein synthesis, maturation and secretion (Sparkes et al., 2009; Eichmann and Schafer, 2012). Adverse environmental stresses can impact this ER quality control (ER QC) machinery causing unfolded/misfolded proteins to accumulate in the ER. Perturbations in ER function are identified by transmembrane sensors which activate signal transduction that increases gene expression of ER resident chaperones and foldases. Collectively, this is known as the unfolded protein response (UPR). The UPR orchestrates restoration of ER homeostasis and protein production by: (i) inducing expression of chaperones and foldases to facilitate protein folding, (ii) translational repression to reduce the protein load on the ER and (iii) removal of unfolded proteins from the ER for degradation via the proteasome (Liu and Howell, 2010b).

In addition to protein synthesis and maturation, the ER also serves as the primary reservoir for intracellular Ca^+^^2^ storage. The Ca^2^^+^ that is concentrated in the ER facilitates the activity of ER resident chaperones and foldases. The mitochondria influences Ca^2^^+^ release from the ER as well as returning Ca^2^^+^ during a recovery phase (Berridge, 2002). Thus the ER and mitochondria cooperate to generate a continuous, tightly regulated Ca^2^^+^ signaling pathway. Moreover, ER stress is one stimulus that can trigger the release of Ca^2^^+^ from the ER into the mitochondria resulting in diminished protein folding capacity in the ER.

## MAMMALIAN RESEARCH HAS PROVIDED A FRAMEWORK LINKING ER STRESS AND PROGRAMMED CELL DEATH

In mammals, chronic stress on the ER can promote oxidative stress, autophagy, and apoptotic cell death. In plants, the UPR is also linked to oxidative stress and PCD regimes that include autophagy. Cell death is seen as an adaptive and intended response to reinforce a system that is overwhelmed. Thus, in situations where the demand for protein and/or Ca^+^^2^ outweighs a given cells ability to cope, prolonged ER stress leads to harsher measures and activates a PCD that, in mammals, can be either autophagous and/or apoptotic (Hetz, 2012; Jager et al., 2012). In this review we focus on ER-directed cell death pathways in plants and discuss; (i) signal transduction mediating ER-stress induced cell autophagic/programmed cell death; (ii) the role of ER resident chaperones in suppressing PCD, (iii) the regulation/crosstalk of ER-induced PCD and autophagy pathways, and (iv) ER-stress regulated PCD during plant pathogen interactions.

### ER SENSORS CONTROL STRESS RESPONSES AND CELL DEATH PATHWAYS

The detection of ER stress and mediation of UPR signaling occurs via sensors located at the ER membrane. In mammals there are three transmembrane embedded sensors: IRE1, ATF6, and PERK (Walter and Ron, 2011). Each of these sensors initiate signaling pathways that can restore ER homeostasis or under conditions of chronic stress and increasing damage, activate alternative routes leading to cell death. The type 1 transmembrane protein, kinase/endoribonuclease inositol-requiring enzyme 1 (IRE1 a and b), is a dual functioning protein. IRE1 has ribonuclease activity and is responsible for splicing X-box binding protein-1 (XBP) mRNA, generating the transcription factor XBP1 (Yoshida et al., 2001). XBP1 translocates to the nucleus where it activates the expression of cytoprotective genes, including members of the ER QC pathway. IRE1 is also capable of activating the apoptotic-signaling kinase 1 (ASK1) and Jun-N-terminal kinase (JNK) that promote apoptosis (Koizumi et al., 2001; Nagashima et al., 2011; Humbert et al., 2012).

In plants, IRE1a and IRE1b localize to the perinuclear ER and the signaling pathways that emanate from these sensors include a completely separate set of intermediary factors from those identified in mammals (Koizumi et al., 2001). In fact recent data suggest a distinct and specialized role for IRE1b in ER stressed but not nutrient stressed induced autophagy and further suggests that an alternative pathway is in play during autophagy responses during nutrient deprivation in plants (Liu et al., 2012).

IRE1 activates mRNA splicing of the transcription factor, bZIP60 which recognizes promoters with a recently identified ER stress responsive *cis*-element UPRE-III in the NAC103 promoter. The NAC103 transcription factor activates several UPR related foldases including CRT1, CNX, PDI-5 (Sun et al., 2013a). Both IRE1 proteins have been reported to be expressed throughout the plant (Noh et al., 2002). While autophagy markers including the formation of autophagosomes were observed following nutrient starvation, wild type, IRE1a and IRE1b mutants resulted in similar phenotypes; (Liu et al., 2012). Treatment of *Arabidopsis* IRE1a and IRE1b knock out mutants with the well-known ER stress inducers, tunicamycin (TM) and dithiothreitol (DTT), resulted in contrasting phenotypes. Application of DTT and TM induced the formation of autophagosomes in IRE1a mutants and wild type plants. In contrast, autophagy was not induced in IRE1b mutants under the same conditions (Liu et al., 2012). ER stress-induced autophagy in plants occurs only via the IRE1b-mediated pathway (Koizumi et al., 2001; Liu et al., 2012).

In mammals, ATF6 is a type II transmembrane basic leucine-zipper (bZIP) domain-containing activating transcription factor (Yoshida et al., 2001). Upon ER stress ATF6 moves through the Golgi compartment and is cleaved by cellular proteases for maturation. The plant equivalents to the mammalian ATF6 pathway, are two key ER-localized, membrane tethered transcription factors, bZIP17 and bZIP28 (Liu et al., 2007a,b). Similar to ATF6, bZIP17, and bZIP28 are activated following detection of accumulating unfolded proteins in the ER and then translocate to the Golgi apparatus where they are cleaved by Golgi-localized proteases for maturation. In the nucleus bZIP17 and bZIP28 activate expression of cytoprotective chaperones and foldases, facilitating the formation of correct macromolecular structures and protein folding, respectively (Liu et al., 2007a, 2008; Liu and Howell, 2010a; Srivastava et al., 2012).

The third ER resident sensor identified in mammals is the type I transmembrane protein kinase RNA-like ER kinase (PERK). Upon detection of unfolded proteins and ER stress, PERK phosporylates and inactivates the translation initiation factor eIF2a to shut down protein synthesis (Harding et al., 2000). PERK also activates the transcription factor CHOP which induces gene expression that leads to apoptosis. Translational regulation may not be entirely conserved since no obvious PERK homologs have as yet been identified in plants (Urade, 2009; Eichmann and Schafer, 2012).

Calcium stores in the ER are critical for the functioning of certain ER resident foldases. Calcium imbalance in the ER can disrupt the functioning of this protein folding pathway causing malformed proteins to accumulate. Release of Ca^+^^2^ from the ER can interfere with protein folding and leads to increased Ca^2^^+^ levels in the mitochondria and can promote oxidative stress and ultimately cell death (Berridge, 2002). Given the critical role of calcium in protein folding, oxidative stress and programmed cell death, mammalian systems utilize several different regulators including Bcl-2 and family members (Bax, and Bak), which are cytoprotective calcium sensors that modulate the release of ER Ca^+^^2^ stores and regulate cell death. Given the importantance of calcium in protein folding in the plant ER, it is in some ways surprising that genome sequence comparisons between plants and mammals indicate that these Bcl-2 family members are not present in plants, at least at the level of primary DNA sequence. Remarkably, transgenically expressed cytoprotective Bcl-2 and others (e.g., nematode Ced-9, chicken Bcl-xl; insect IAP; viral p35) function in plants in a similar manner to what occurs in animals including inhibiting PCD in response to pathogen invasion and abiotic/environmental stresses, in accordance with transkingdom pathway conservation (Dickman et al., 2001; Lincoln et al., 2002; Williams and Dickman, 2008; Dickman and Fluhr, 2013). Thus mammalian anti-apopotic machinery functions in plants and points to a conserved apoptotic-like PCD mechanism for death in plants. A key issue that is not entirely reconciled, is that there is little evidence for conservation at the DNA/gene level. We have suggested that structural homologies independent of sequence account for functional conservation, and indeed we have shown this to be the case when we uncovered the *Arabidopsis* BAG gene family (Doukhanina et al., 2006; Kabbage and Dickman, 2008; Williams et al., 2010). Initial blast analyses of *Arabidopsis* nucleotide and amino acid sequences failed to identify homologs of the mammalian BAG family. Therefore more sensitive methods were used based on higher level conservation including Hidden Markov Modelling (HMM) and profile-profile alignment algorithims to identify seven BAG members in Arabidopsis (Doukhanina et al., 2006). Unlike animals, plant BAGs display unique sub-cellular localisation; the three predicted calmodulin-binding BAGs are localized in the ER, mitochondria and vacuole, all of which are known Ca^2^^+^ reservoirs and mediators of cell death pathways (Kabbage and Dickman, 2008; Williams et al., 2010; Dickman and Fluhr, 2013).

## WHEN PROTEINS FAIL TO FOLD- PROTEIN DEGRADATION OR SELF-DESTRUCTION?

**FIGURE 1:**
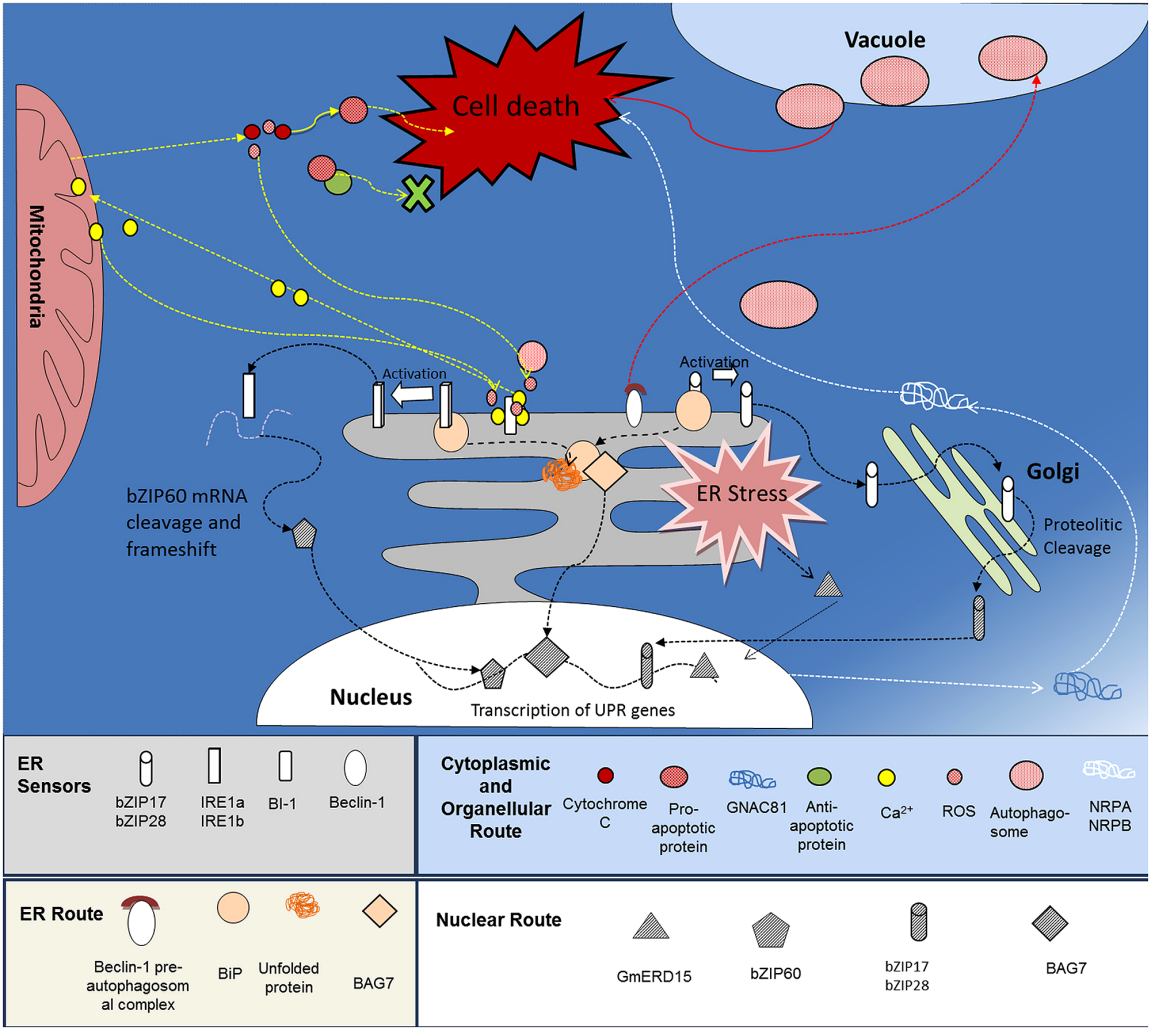
**ER-mediated cell death pathways in plants.** The ER and ER stress pathways are key molecular switches of at least three routes leading to cell death, (i) intracellular signaling pathways - ROS triggers the mobilization of Ca^2^^+^ from the ER to the Mitochondria, resulting in a mitochondrial permeability transition pore (PTP), selective leakage of apoptotic factors, additional release of Ca^2^^+^ from the ER and cell death (Yellow arrows). (ii) ER-vacuole directed autophagy - ER-localized Beclin-1 initiates autophagosome formation, autophagy, and cell death, and (iii) NRP-directed cell death - detection of ER and osmotic stress activates the transcription factor GmERD15 which triggers expression of NRPA and -B. Once expressed, the NRPs activate the cell death effector GmNAC81 leading to DNA fragmentation and cell death (white arrows).

There are occasions when proteins fail to mature in the ER and as a result, they are exported from the ER for degradation by the ubiquitin-proteasome system as part of the endoplasmic reticulum assisted degradation (ERAD) pathway (Liu and Howell, 2010b; Huttner and Strasser, 2012). When plants are subjected to environmental stress, the levels of malformed proteins can overwhelm the ERQC and the ERAD associated systems. Continuing ER stress requires harsher measures including oxidative stress that can lead to autophagy or apoptosis in mammals (Hetz, 2012; Jager et al., 2012; **Figure 1**). How these life/death decisions are made; where is the point-of no return from commitment to death; how these options are distinguished, identification of the key regulatory and the nature of PCD pathways promises to be of continuing interest.

Autophagy involves the sequestration of unwanted or damaged proteins and organelles in characteristic double membrane vesicles known as autophagosomes. There are more than 30 autophagy (ATG) related genes in yeast, many of which have been identified in mammals and plants. The mechanism of autophagosome formation has been described elsewhere but it is important here to recognize that the ER is active in the formation of these double membrane vesicles (Axe et al., 2008). Autophagosomes engulf cytosolic constituents and/or damaged organelles then deliver their “cargo” to either the vacuole (plants) or lysosome (mammals) where they are degraded. Autophagy is often viewed as a drastic measure taken by cells to eliminate damaged proteins or organelles with the goal of restoring cellular homeostasis, as first noticed during conditions of yeast starvation (Takeshige et al., 1992).

Autophagy is involved with the UPR via the IRE1a pathway in mammals (Sano and Reed, 2013). In plants, autophagy is linked to IRE1b but splicing of bZIP60 mRNA is not required for autophagy. This dispensability of IRE1b splicing of bZIP60 suggests one of two possibilities; IRE1b may have an additional, as yet unknown splicing target that is linked to activation of autophagic machinery (Liu et al., 2012), or IRE1b may have additional functions (e.g., kinase activity) which activate an alternate signaling cascade.

Several environmental, nutritional, pathogenic and metabolic conditions including those that promote ER stress have been linked with autophagy. Thus a growing body of evidence suggests that autophagy can play opposing roles by promoting both cell survival and cell death (Liu et al., 2005; Hayward and Dinesh-Kumar, 2011). Moreover, as the ER is also crucial for maintenance of cellular homeostasis under stress, autophagy mediated trafficking of nutrients and removal of “damaged goods” can provide the cell necessary time for ER-stress adaptation. Thus, during times of excessive ER stress, autophagy may act as a cellular backup to ERAD bolstering UPR and ERAD capacity in addition to providing essential nutrients to penurious cells (Yorimitsu et al., 2006).

### ER AND PLANT PCD

Programmed cell death is essential for normal growth and development (Kerr et al., 1972), however, disease occurs when PCD is inappropriately regulated (too much, too little). In mammals this can lead to a range of pathologies including cancer to Parkinson’s disease. The UPR, autophagy and Ca^2^^+^ signaling between the ER and mitochondria are core components of the PCD machinery. These core components have recently been identified in plants thus linking the UPR to PCD and innate immunity (Ma and Berkowitz, 2007; Martinon and Glimcher, 2011; Stael et al., 2012).

In plants there are four key ER resident factors that link Ca^+^^2^ stores to UPR and cell death regulation. These are calreticulin, calmodulin, ER-type IIA Ca^2^^+^ pump, and type IIB Ca^+^^2^-ATPase. Calreticulin is a Ca^+^^2^ binding protein that chaperones maturation of glycoproteins in the ER (Jia et al., 2009). Calmodulin is a Ca^+^^2^ sensor that recognizes changes in Ca^+^^2^ levels in the ER and activates immune related PCD (Ranty et al., 2006; Ma et al., 2008; Du et al., 2009). Application of the ER-type IIA Ca^2^^+^ pump inhibitor cyclopiazonic acid (CPA) leads to increased cytosolic Ca^2^^+^, cross-talk with mitochondria and PCD in soybean (Zuppini et al., 2004). The formation of the so-called mitochondrial permeability transition pore (PTP), an established marker of PCD, allows the selective leakage of several stress signals and cell death regulators sensitizing ER channels to further release of Ca^2^^+^ in a positive feedback loop (Berridge, 2002). In addition, the ER localized type IIB Ca^+^^2^-ATPase regulates N-mediated PCD in response to infection with Tobacco mosaic virus (TMV; Zhu et al., 2010). Perturbations in ER function, however, promote the release of Ca^2^^+^ from the ER into the mitochondria resulting in diminished protein folding capacity, ER stress, the formation of PTP and subsequent apoptosis (Berridge, 2002).

In addition to disruption of ER function and the accumulation of unfolded proteins, studies have shown that depletion/release of Ca^2^^+^ from the ER is promoted by pro-death members of the Bcl-2 family (Scorrano et al., 2003; Hammadi et al., 2013). Additionally, cytoprotective anti-PCD proteins such as Bcl-2 have been shown to prevent PCD by suppressing the release ER Ca^2^^+^ (Lam et al., 1994). One protein that may facilitate ER Ca^2^^+^ homeostasis by acting as a Ca^2^^+^ permeable channel is Bax inhibitor and is discussed below.

A plant-specific branch of the ER stress pathway was recently noted in soybean; N-rich protein (NRP)-mediated cell death. NRPs are asparagine rich proteins that represent the hallmarks of an integrative pathway linking ER and osmotic stress signaling (Reis and Fontes, 2012). The combination of ER-stress and osmotic stress activates the transcription factor GmERD15 which in turn binds to and triggers expression of N-rich protein genes including NRP-A and NRP-B (Reis and Fontes, 2012). Overexpression of these NRPs in soybean induces the cell death effector GmNAC81, activating a caspase-3-like activity promoting DNA fragmentation; features associated with PCD. The ER chaperone BiP limits spread of this death signal (Reis et al., 2011). In a yeast two-hybrid screen for GmNAC81 interactors, GmNAC30 was identified. Interestingly, a functionally relevant binding site for GmNAC81/ GmNAC30 was found in the promoter of vacuolar processing enzyme (VPE; Mendes et al., 2013). VPE is a plant protease the activity of which, is important for viral and bacterial-induced HR. VPEs have been reported on several occasions to induce PCD in plants following pathogen challenge. Although structurally unrelated to caspases, VPE has caspase-1 (an inflammatory caspase) activity (Hatsugai et al., 2004; Hara-Nishimura et al., 2005; Kuroyanagi et al., 2005). This system links ER stress, the UPR, and cell death regulation. Transgenic tobacco plants overexpressing BIP show enhanced water stress tolerance (Alvim et al., 2001; Valente et al., 2009), thus there is a potential biotechnological application by generating crop plants (e.g., soybean) overexpressing BiP that would mitigate ER stress and lead to enhanced water stress tolerance. Interestingly, NRP signaling is believed independent of the known ER resident stress sensor mechanisms; thus it will be of particular interest to identify key components and piece together these PCD pathway(s).

## ROLE OF THE ER RESIDENT CHAPERONES *BAG-7, BIP* AND *BI-1* IN LIMITING CELL DEATH

### BAG FAMILY OF PROTEINS

Members of the Bcl-2-associated athanogene (BAG) family are a conserved group of eukaryotic co-chaperones that assist in protein folding and diverse cellular processes including apoptosis and stress signaling. The *Arabidopsis* BAG family contains seven members and unlike its mammalian counterparts which are primarily cytosolic, different family members localize to different sub-cellular organelles (Doukhanina et al., 2006; Kabbage and Dickman, 2008; Williams et al., 2010). BAG proteins regulate Bcl-2, interact with Hsp70/Hsc70, and associate with the ubiquitin/proteasome systems in animals. BAG proteins function in processes that promote cell survival in both plants and animals (Kabbage and Dickman, 2008).

In the ER, AtBAG7 plays a key role in UPR pathways, particularly in response to heat and cold stress (Williams et al., 2010). AtBAG7 directly binds to the ER-resident sensor and chaperone AtBiP2 (Williams et al., 2010) and likely provides essential co-chaperoning activity needed to prevent the build-up of unfolded proteins and limit cell death as a result. Interestingly, AtBAG7 mutants display hypersensitivity to the ER-stressor and autophagy inducer TM, suggesting that AtBAG7 plays a role in the regulation of autophagy pathways (Williams et al., 2010). Moreover such mutants were also sensitive to the ER stress stimuli heat and cold.

Subsequent staining of TM treated wild type and AtBAG7 mutant plants with the autophagosome detecting dye monodansylcadaverine (MDC) however, showed autophagosomes are formed in the presence or absence of AtBag7. Thus, AtBag7 does not appear to be necessary for autophagy. Our preliminary data indicates that AtBAG7 translocates from the ER to the nucleus in response to heat stress. Yeast two hybrid and BIFC studies show that following nuclear translocation, AtBAG7 binds to the transcription factor BTF3, a member of the NAC family of stress responsive transcription factors. It is reasonable to hypothesize that ER-localized AtBAG7 activates stress response pathways via translocation to the nucleus and transcriptional activation of genes associated with abiotic stress protection. In this respect AtBAG7 may not only play a role as an important ER co-chaperone but may be an important mediator of ER – nucleus signaling during ER stress responses. Studies are in progress to determine the contributions of AtBAG7 to the UPR and PCD.

### BiP

The ER luminal binding protein (BiP/Grp78) is a member of the heat shock protein 70 (HSP70) family (Haas, 1994). It is the most abundant ER-chaperone, and a key player of the ERQC machinery. BiP binds to and suppresses the activity of the mammalian ER stress sensors, PERK, IRE1, and ATF6 and the plant ER stress sensors IRE1, bZIP17, and bZIP28 (Srivastava et al., 2013). Upon detection of unfolded proteins, however, BiP is released and binds to unfolded proteins thus leaving IRE1 free to oligomerise and splice bZIP60 mRNA, the result of which induces transcription of UPR associated genes.

Three BiP isoforms have been identified in *Arabidopsis* (Lin et al., 2001). Expression of BiP is induced by environmental stressors such as heat, salinity and pathogen invasion (Sung et al., 2001). Expression profiling has shown that BiP 1 and 2 are constitutively expressed while activation of BiP3 is triggered by bZIP60 as part of the UPR (Iwata et al., 2008; Deng et al., 2011). BiP3 expression is also induced in the absence of bZIP60, suggesting additional factors mediate transcription of BiP3 in the IRE/bZIP60 pathway. As mentioned BiP mediated inhibition of UPR is a factor in drought in transgenic tobacco (Alvim et al., 2001). BiP is also a negative regulator of ER stress related cell death during pathogen invasion (Jelitto-Van Dooren et al., 1999). Surface exposed leucine rich receptors are responsible for recognition of the bacterial pathogen-associated molecular patterns (PAMPs) and are critical in plant defense. Complex formation between BiP, and other ER resident proteins such as stromal-derived factor-2 (SDF-2) and calreticulin is essential for the glycosylation, activity and accumulation of the receptor kinases EFR and Cf-9, respectively. Similarly, loss of BiP complex formation causes ER retention and reduced levels of EFR and Cf-9 (Nekrasov et al., 2009; Liebrand et al., 2012). Taken together, in addition to reducing unfolded protein levels, BiP stabilizes immune receptors to facilitate host defense.

### BAX INHIBITOR (BI-1)

Initially identified as a suppressor of BAX-induced (BI) cell death in yeast and mammalian cells, Bax-inhibitor proteins are highly conserved ER localized, multi-transmembrane proteins (Ishikawa et al., 2011). BI-1 genes are one of the few examples of a gene identified and characterized in both animals and plants that specifically regulate PCD. There is increasing evidence that BI-1 plays roles in both PCD and autophagy to regulate the switching of ER-mediated cell death pathways from autophagy to PCD (Castillo et al., 2011; Carvalho et al., 2013).

Over-expression of BI-1 reduces PCD as well as the cytosolic Ca^2^^+^ concentration, potentially by functioning as a pH sensitive regulator of ER calcium channel activity. Evidence for these functions is supported by topology studies which indicate that the C-terminus of BI-1 forms a Ca^2^^+^ pore that could act as the source for its Ca^2^^+^ -leaking properties (Bultynck et al., 2012). BI-1 also plays a role in autophagy responses via interaction with the ER stress sensor, IRE1; via interaction with BI-1, IRE-1 is activated leading to induction of UPR gene expression (Lisbona et al., 2009).

As in mammals *Arabidopsis*/plant Bax Inhibitor-1 (AtBI-1) also suppresses Bax when expressed in tobacco, as well as several abiotic and biotic stress-induced PCD (Watanabe and Lam, 2006, 2008). AtBI-1 mutants also display increased sensitivity to ER stress factors such as heat and TM. Conversely, *Arabidopsis* plants over expressing AtBI-1 are more tolerant to TM, heat and cold stress (Watanabe and Lam, 2008). Studies have indicated that BI-1 functions downstream of ROS generation; over-expression of BI-1 in *Arabidopsis* did not suppress BAX-induced ROS production but still prevented PCD (Kawai-Yamada et al., 2001, 2004). AtBI-1 prevents Bax-induced cell death at least in part, by maintaining ER Ca^2^^+^ homeostasis (Ihara-Ohori et al., 2007). AtBI-1 knockouts display accelerated methyl jasmonate-induced senescence pathways further linking the ER to autophagy (Yue et al., 2012).

A role for BI-1 in the regulation of plant autophagy responses is also suggested by starvation experiments. Carbon starvation is a known inducer of autophagy in plants and animals; down-regulation of the tobacco BI-1 homolog augments cell death upon starvation (Bolduc and Brisson, 2002). Importantly, *Arabidopsis* BI-1 knockouts did not display any developmental abnormalities suggesting a specific role for BI-1 during these stress conditions.

## DEATH BY DESIGN? THE ROLE OF BECLIN-1 AND BCL-2 IN ER-INDUCED AUTOPHAGY AND PCD

Prolonged ER stress and/or excessive autophagy may induce PCD; paradoxically, autophagy mutants display increased PCD during nitrogen starvation. Thus either too much or too little autophagy can lead to PCD. The relationship and cellular decision process mediating autophagy vs. PCD is largely unknown. During the initial stages of stress, autophagy pathways may be triggered to reduce ER stress and prevent apoptosis. As a result homeostasis is maintained but whether the default state of autophagy is pro-survival or pro-death (or both) remains to be elucidated, as there are examples of both situations (Hofius et al., 2009, 2011; Xu et al., 2013) As in the case of N-gene mediated resistance to TMV, pro-survival roles for autophagy in cytoprotective cell death, include the “controlled sacrifice” of select cells and organelles providing nutritional building blocks to the organism/cell, maintaining energy homeostasis during a potentially lethal situation. Importantly, for N-gene mediated resistance to tobacco mosaic virus, autophagy is needed to limit the extent of cell death surrounding the cells attacked by the virus (Liu et al., 2005). If autophagy is blocked, disease symptoms are no longer constrained. In other situations, sustained autophagy triggers apoptosis in mammals and in this case cell death may not be cytoprotective (Rikiishi, 2012). In this respect autophagy may be considered as a last ditch effort for cell survival, once the irreversible apoptotic cell decision is made, death is inevitable. The salient details as to how and when these life-death decisions are determined, also remain to be established. In mammals the interaction between Bcl-2 and Beclin-1 functions as a switch that drives the direction of signaling either toward autophagy or apoptosis (Oberstein et al., 2007).

The Bcl-2 family contains both pro-apoptotic proteins (e.g., BAX) and anti-apoptotic proteins including Bcl-2 (Reed, 1998). Family members are distinguished by 1-4 so-called BH (Bcl-2 homology domains). BH3 domains are of particular importance as all pro-apoptotic family members harbor this signature “death” domain required for apoptosis. Anti-apoptotic proteins inhibit cell death by binding to these domains. Beclin-1 (ATG6) is a conserved BH3 domain containing protein and primary component of the autophagy pathway that was initially identified as a Bcl-2 interacting protein. Bcl-2, if in sufficient concentration inhibits Beclin-1 mediated autophagy by binding to the BH3 domain in Beclin-1, thereby removing free Beclin-1. Thus Bcl-2 is not only anti-apoptotic, but also anti-autophagic. If Beclin-1 is in excess, Bcl-2 is tied up and autophagy ensues. Thus Beclin-1 serves as a checkpoint dictating the balance between apoptosis and autophagy pathways. The BH3 domain of Beclin-1 interacts with several anti-apoptotic Bcl-2 family members (Maiuri et al., 2010). Once bound, Beclin-1 is unable to assemble the pre-autophagosome complex thereby inhibiting autophagy while promoting apoptosis (Sinha and Levine, 2008). Plant Beclin-1 appears functionally and structurally similar to mammalian Beclin-1 (Sinha and Levine, 2008) with a notable difference; plant Beclin-1 lacks the mammalian BH3 death domain. Is there a functional or structural equivalent? What are the underlying mechanisms of Beclin-1 mediated autophagy and/or PCD?

## ER-CELL DEATH PATHWAYS AND PLANT PATHOGEN INTERACTIONS

Recent research has begun to identify links between plant pathogens and ER stress related cell death. Studies with *Potato virus X* (PVX) show that the ER residing PVX TGBp3 movement protein activates the transcription factor bZIP60 to trigger the UPR as well as eliciting PCD (Ye et al., 2012, 2013). The mechanism appears to be conserved across host species, with expression of TGBp3 inducing the same sets of genes in *Arabidopsis thaliana* and *Nicotiana benthamiana*. *Tobacco rattle virus* mediated knockdown of the bZIP60 pathway significantly blocked accumulation of PVX in both protoplasts and whole plants; thus activation of bZIP60 is required for PVX replication. Additionally, infiltration of *Agrobacterium* carrying a TGBp3 expression system induced an HR in tobacco that could be abrogated by co-expression of BiP but not by anti-apoptosis genes Bcl-xl, Ced-9, Op-IAP. Such a hypersensitive response was not elicited upon infiltration of either TMV or PVX, thus indicating that TGBp3 elicits PCD but also the UPR for survival (Ye et al., 2013).

Reoviruses are double-stranded RNA viruses and infect a wide range of eukaryotes. Rice black-streak dwarf virus (RBSDV) P10 outer capsid protein induces ER stress and the UPR in *N*. *benthamiana*. Similar to the PVX TGBp3 protein, P10 associates with the ER and induces expression of bZIP60, BIP, PDI, and Calmodulin (CAM). Mammalian infecting reoviruses are also known to elicit the UPR to benefit virus replication and assembly. These viruses can sequester pro-death factors inside viroplasms to regulate antiviral defenses that could radiate from UPR initiation. These data suggest that virus interactions with the UPR machinery are conserved across eukaryotes and offer some unique perspectives on how viruses could control PCD via their interaction with the ER (Zambrano et al., 2011; Sun et al., 2013b).

Endoplasmic reticulum resident chaperones, BiP and calreticulin (CRT) have also been demonstrated to play a key role in resistance against the vascular wilt necrotrophic fungal pathogen *Verticillium dahliae*. Tomato Ve1, an LRR receptor-like protein confers resistance in tomato to *V. dahliae*. In a screen employing GFP fusions, immuno-screening and mass spectrometry, potential binding partners with Ve1 were identified including several ERQC chaperones including BiP and a lectin-like calreticulin (CRT) (Liebrand et al., 2014). Knockdown of tomato BiPs and CRT in the tomato plants carrying the Ve1 gene resulted in reduced resistance to *Verticillium* suggesting that both ERQC chaperones contribute to plant immunity. Interestingly, parallel experiments in tobacco and *N. benthamiana* failed to detect changes in glycosylation on Ve1 and unlike non-silenced tomato there was no suppression of HR, even though plants were more susceptible in the Ve1 CRT silenced tobacco lines. This suggests that a HR is not required and/or could indicate an uncoupling between cell death and resistance. An interesting example of ER targeting for compatibility was elegantly demonstrated in studies with *Piriformospora indica*, a fungal mutualist (Qiang et al., 2012). For successful root colonization *P*. *indica* initially colonizes living cells. During this period the fungus triggers ER stress but inhibits the UPR. Of note, VPE was also induced and mediated ER stress and cell death in large part by suppressing the host (*Arabidopsis*) UPR pathway. A similar scenario of colonizing living cells while preparing for their doom via ER stress regulation might be occurring in *Verticillium* as described above as well as the necrotroph *Sclerotinia sclerotiorum. Sclerotinia* is an aggressive, broad host range necrotroph that was recently shown to also colonize living cells prior to fungal induced host cell death. Sclerotinia oxalic acid mutants are non-pathogenic and elicit in the host a bona fide HR and autophagy (Kabbage et al., 2013). Treatment of this non-pathogenic mutant with DTT partially restored pathogenicity suggesting that the fungus may require ER stress control for successful infection (Williams et al., 2011).

Studies using differential lines of barley to the powdery mildew fungus *Blumeria graminis* f.sp. *hordei* (Bgh) suggest a functional role for BI-1, the ER and UPR in response to fungal challenge (Huckelhoven et al., 2003). Several lines of evidence suggest an inverse relationship between BI-1 function and host penetration resistance against the powdery mildew fungus. Overexpression of barley BI-1 increased susceptibility to Bgh. Barley BI-1 expression is significantly suppressed following application of the salicylic acid analog, 2,6-dichloroisonicotinic acid (INA), an inducer of systemic resistance (Huckelhoven et al., 2003) Together the cell death suppressing activity of cytoprotective BI-1, compromises host defense mechanisms that generate a PCD –HR for resistance, providing a link between PCD host defense pathways and BI-1 expression.

## CONCLUSION

We are just beginning to uncover the signaling pathways and regulatory circuits mediating ER stress and cell death in plants. Oxidative stress, Ca^+^^2^ influx to mitochondria, caspase-like activities, autophagy, and PCD related factors contribute to the ER stress response.

Cells appear to implement a hierarchal regime involving a series of checks and balances before succumbing to ER cell death. Prolonged ER-stress leads to oxidative stress and if sustained; autophagy can occur. If autophagy is not sufficient to right the ship, PCD removes the cell. Autophagy mutants display increased PCD during nitrogen starvation; thus too much or too little autophagy can result in PCD. Beclin-1 appears to serve as a key checkpoint illustrating the intricate balance between pro-survival and pro-death within autophagy pathways. By default, autophagy pathways appear to be pro-survival and can be considered a last ditch effort by the organism to cope with prolonged stress and prevent the “point of no return” that leads to the induction of apoptosis pathways. Intriguingly, although, Bcl-2 family members have not been found in plants, mammalian BCL-2 family members have been expressed in plants and demonstrate conserved function transkingdom manner, thus suggesting that structural /functional homologs of the Bcl-2 family exist in plants (Dickman et al., 2001; Doukhanina et al., 2006). The absence of a BH3 domain in plant Beclin-1 correlates with the failure to identify Bcl-2 family members and the question remains open as to whether there is a functionally operationally conserved mechanism or not in plants.

The ER and ER stress pathways are becoming increasingly more prominent as potential targets for the pathogenic success of microbial pathogens. We anticipate this to continue. For example, the fungus *P. indica* induces cell death by inhibiting the UPR related pro-survival machinery and then activating ER stress mediated cell death machinery (Qiang et al., 2012). Moreover, numerous plant viruses commandeer ER stress machinery to mitigate host defense and the HR further highlighting the ER as a master switch in biotic (and abiotic) environmental stresses.

Key future issues include: (i) Identifying the relevant players in plants and filling in the gaps in the in ER stress signaling pathways and (ii) Deciphering ER mediated cell decision processes. Although there are gaps in our knowledge surrounding ER-stress induced cell death pathways, it is apparent that the ER and UPR form a tight “cell death” regulatory network with several plant organelles that together facilitate homeostasis in mammals and plants in response to development and environmental cues.

## Conflict of Interest Statement

The authors declare that the research was conducted in the absence of any commercial or financial relationships that could be construed as a potential conflict of interest.

## References

[B1] Alvim F. C. Carolino S. M. Cascardo J. C. Nunes C. C. Martinez C. A. Otoni W. C. (2001). Enhanced accumulation of BiP in transgenic plants confers tolerance to water stress. *Plant Physiol.* 126 1042–1054 10.1104/pp.126.3.1042 11457955 PMC116461

[B2] Axe E. L. Walker S. A. Manifava M. Chandra P. Roderick H. L. Habermann A. (2008). Autophagosome formation from membrane compartments enriched in phosphatidylinositol 3-phosphate and dynamically connected to the endoplasmic reticulum. *J. Cell Biol.* 182 685–701 10.1083/jcb.200803137 18725538 PMC2518708

[B3] Berridge M. J. (2002). The endoplasmic reticulum: a multifunctional signaling organelle. *Cell Calcium* 32 235–249 10.1016/S0143416002001823 12543086

[B4] Bolduc N. Brisson L. F. (2002). Antisense down regulation of NtBI-1 in tobacco BY-2 cells induces accelerated cell death upon carbon starvation. *FEBS Lett.* 532 111–114 10.1016/S00145793(02)03650-5 12459473

[B5] Bultynck G. Kiviluoto S. Henke N. Ivanova H. Schneider L. Rybalchenko V. (2012). The C terminus of Bax inhibitor-1 forms a Ca2+-permeable channel pore. *J. Biol. Chem.* 287 2544–2557 10.1074/jbc.M111.275354 22128171 PMC3268414

[B6] Carvalho H. H. Silva P. A. Mendes G. C. Brustolini O. J. Pimenta M. R. Gouveia B. C. (2013). The endoplasmic reticulum binding protein BiP displays dual function in modulating cell death events. *Plant Physiol.* 164 654–670 10.1104/pp.113.231928 24319082 PMC3912096

[B7] Castillo K. Rojas-Rivera D. Lisbona F. Caballero B. Nassif M. Court F. A. (2011). BAX inhibitor-1 regulates autophagy by controlling the IRE1alpha branch of the unfolded protein response. *EMBO J.* 30 4465–4478 10.1038/emboj.2011.318 21926971 PMC3230375

[B8] Deng Y. Humbert S. Liu J. X. Srivastava R. Rothstein S. J. Howell S. H. (2011). Heat induces the splicing by IRE1 of a mRNA encoding a transcription factor involved in the unfolded protein response in *Arabidopsis*. *Proc. Natl. Acad. Sci. U.S.A.* 108 7247–7252 10.1073/pnas.1102117108 21482766 PMC3084119

[B9] Dickman M. B. Fluhr R. (2013). Centrality of host cell death in plant-microbe interactions. *Annu. Rev. Phytopathol.* 51 543–570 10.1146/annurev-phyto-081211-173027 23915134

[B10] Dickman M. B. Park Y. K. Oltersdorf T. Li W. Clemente T. French R. (2001). Abrogation of disease development in plants expressing animal antiapoptotic genes. *Proc. Natl. Acad. Sci. U.S.A.* 98 6957–6962 10.1073/pnas.091108998 11381106 PMC34460

[B11] Doukhanina E. V. Chen S. Van Der Zalm E. Godzik A. Reed J. Dickman M. B. (2006). Identification and functional characterization of the BAG protein family in *Arabidopsis thaliana*. *J. Biol. Chem.* 281 18793–18801 10.1074/jbc.M511794200 16636050

[B12] Du L. Ali G. S. Simons K. A. Hou J. Yang T. Reddy A. S. (2009). Ca(2+)/calmodulin regulates salicylic-acid-mediated plant immunity. *Nature* 457 1154–1158 10.1038/nature07612 19122675

[B13] Eichmann R. Schafer P. (2012). The endoplasmic reticulum in plant immunity and cell death. *Front. Plant Sci.* 3:200. 10.3389/fpls.2012.00200 PMC3424470 22936941

[B14] Haas I. G. (1994). BiP (GRP78), an essential hsp70 resident protein in the endoplasmic reticulum. *Experientia* 50 1012–1020 10.1007/BF01923455 7988659

[B15] Hammadi M. Oulidi A. Gackiere F. Katsogiannou M. Slomianny C. Roudbaraki M. (2013). Modulation of ER stress and apoptosis by endoplasmic reticulum calcium leak via translocon during unfolded protein response: involvement of GRP78. *FASEB J.* 27 1600–1609 10.1096/fj.12-218875 23322163

[B16] Hara-Nishimura I. Hatsugai N. Nakaune S. Kuroyanagi M. Nishimura M. (2005). Vacuolar processing enzyme: an executor of plant cell death. *Curr. Opin. Plant Biol.* 8 404–408 10.1016/j.pbi.2005.05.016 15939660

[B17] Harding H. P. Novoa I. Zhang Y. Zeng H. Wek R. Schapira M. (2000). Regulated translation initiation controls stress-induced gene expression in mammalian cells. *Mol. Cell.* 6 1099–1108 10.1016/S1097-2765(00)00108-8 11106749

[B18] Hatsugai N. Kuroyanagi M. Yamada K. Meshi T. Tsuda S. Kondo M. (2004). A plant vacuolar protease, VPE, mediates virus-induced hypersensitive cell death. *Science* 305 855–858 10.1126/science.1099859 15297671

[B19] Hayward A. P. Dinesh-Kumar S. P. (2011). What can plant autophagy do for an innate immune response? *Annu. Rev. Phytopathol.* 49 557–576 10.1146/annurev-phyto-072910-095333 21370973

[B20] Hetz C. (2012). The unfolded protein response: controlling cell fate decisions under ER stress and beyond. *Nat. Rev. Mol. Cell Biol.* 13 89–102 10.1038/nrm3270 22251901

[B21] Hofius D. Munch D. Bressendorff S. Mundy J. Petersen M. (2011). Role of autophagy in disease resistance and hypersensitive response-associated cell death. *Cell Death Differ.* 18 1257–1262 10.1038/cdd.2011.43 21527936 PMC3172097

[B22] Hofius D. Schultz-Larsen T. Joensen J. Tsitsigiannis D. I. Petersen N. H. Mattsson O. (2009). Autophagic components contribute to hypersensitive cell death in *Arabidopsis*. *Cell* 137 773–783 10.1016/j.cell.2009.02.036 19450522

[B23] Huckelhoven R. Dechert C. Kogel K. H. (2003). Overexpression of barley BAX inhibitor 1 induces breakdown of mlo-mediated penetration resistance to Blumeria graminis. *Proc. Natl. Acad. Sci. U.S.A.* 100 5555–5560 10.1073/pnas.09314641000931464100 12704231 PMC154383

[B24] Humbert S. Zhong S. Deng Y. Howell S. H. Rothstein S. J. (2012). Alteration of the bZIP60/IRE1 pathway affects plant response to ER stress in *Arabidopsis thaliana*. *PLoS ONE* 7:e39023. 10.1371/journal.pone.0039023 PMC3373542 22701744

[B25] Huttner S. Strasser R. (2012). Endoplasmic reticulum-associated degradation of glycoproteins in plants. *Front. Plant Sci.* 3:67. 10.3389/fpls.2012.00067 PMC3355801 22645596

[B26] Ihara-Ohori Y. Nagano M. Muto S. Uchimiya H. Kawai-Yamada M. (2007). Cell death suppressor *Arabidopsis* bax inhibitor-1 is associated with calmodulin binding and ion homeostasis. *Plant Physiol.* 143 650–660 10.1104/pp.106.090878 17142482 PMC1803746

[B27] Ishikawa T. Watanabe N. Nagano M. Kawai-Yamada M. Lam E. (2011). Bax inhibitor-1: a highly conserved endoplasmic reticulum-resident cell death suppressor. *Cell Death Differ.* 18 1271–1278 10.1038/cdd.2011.59 21597463 PMC3172100

[B28] Iwata Y. Fedoroff N. V. Koizumi N. (2008). *Arabidopsis* bZIP60 is a proteolysis-activated transcription factor involved in the endoplasmic reticulum stress response. *Plant Cell* 20 3107–3121 10.1105/tpc.108.061002 19017746 PMC2613661

[B29] Jager R. Bertrand M. J. Gorman A. M. Vandenabeele P. Samali A. (2012). The unfolded protein response at the crossroads of cellular life and death during endoplasmic reticulum stress. *Biol. Cell* 104 259–270 10.1111/boc.201100055 22268789

[B30] Jelitto-Van Dooren E. P. Vidal S. Denecke J. (1999). Anticipating endoplasmic reticulum stress. A novel early response before pathogenesis-related gene induction. *Plant Cell* 11 1935–1944 10.1105/tpc.11.10.1935 10521523 PMC144106

[B31] Jia X. Y. He L. H. Jing R. L. Li R. Z. (2009). Calreticulin: conserved protein and diverse functions in plants. *Physiol. Plant.* 136 127–138 10.1111/j.1399-3054.2009.1223.x 19453510

[B32] Kabbage M. Dickman M. B. (2008). The BAG proteins: a ubiquitous family of chaperone regulators. *Cell Mol. Life Sci.* 65 1390–1402 10.1007/s00018-008-7535-2 18264803 PMC11131705

[B33] Kabbage M. Williams B. Dickman M. B. (2013). Cell death control: the interplay of apoptosis and autophagy in the pathogenicity of *Sclerotinia sclerotiorum*. *PLoS Pathog.* 9:e1003287. 10.1371/journal.ppat.1003287 PMC3623803 23592997

[B34] Kawai-Yamada M. Jin L. Yoshinaga K. Hirata A. Uchimiya H. (2001). Mammalian Bax-induced plant cell death can be down-regulated by overexpression of *Arabidopsis* Bax Inhibitor-1 (AtBI-1). *Proc. Natl. Acad. Sci. U.S.A.* 98 12295–12300 10.1073/pnas.211423998 11593047 PMC59808

[B35] Kawai-Yamada M. Ohori Y. Uchimiya H. (2004). Dissection of *Arabidopsis* Bax inhibitor-1 suppressing Bax-, hydrogen peroxide-, and salicylic acid-induced cell death. *Plant Cell* 16 21–32 10.1105/tpc.014613 14671021 PMC301392

[B36] Kerr J. F. Wyllie A. H. Currie A. R. (1972). Apoptosis: a basic biological phenomenon with wide-ranging implications in tissue kinetics. *Br. J. Cancer* 26 239–257 10.1038/bjc.1972.33 4561027 PMC2008650

[B37] Koizumi N. Martinez I. M. Kimata Y. Kohno K. Sano H. Chrispeels M. J. (2001). Molecular characterization of two *Arabidopsis* Ire1 homologs, endoplasmic reticulum-located transmembrane protein kinases. *Plant Physiol.* 127 949–962 10.1104/pp.010636 11706177 PMC129266

[B38] Kuroyanagi M. Yamada K. Hatsugai N. Kondo M. Nishimura M. Hara-Nishimura I. (2005). Vacuolar processing enzyme is essential for mycotoxin-induced cell death in *Arabidopsis thaliana*. *J. Biol. Chem.* 280 32914–32920 10.1074/jbc.M504476200 16043487

[B39] Lam M. Dubyak G. Chen L. Nunez G. Miesfeld R. L. Distelhorst C. W. (1994). Evidence that BCL-2 represses apoptosis by regulating endoplasmic reticulum-associated Ca2+ fluxes. *Proc. Natl. Acad. Sci. U.S.A.* 91 6569–6573 10.1073/pnas.91.14.6569 8022822 PMC44244

[B40] Liebrand T. W. H. Kombrink A. Zhang Z. Sklenar J. Jones A. M. E. Robatzek S. (2014). Chaperones of the endoplasmic reticulum are required for Ve1-mediated resistance to *Verticillium.* *Mol. Plant Pathol.* 15 109–117 10.1111/mpp.12071 24015989 PMC6638731

[B41] Liebrand T. W. Smit P. Abd-El-Haliem A. De Jonge R. Cordewener J. H. America A. H. (2012). Endoplasmic reticulum-quality control chaperones facilitate the biogenesis of Cf receptor-like proteins involved in pathogen resistance of tomato. *Plant Physiol.* 159 1819–1833 10.1104/pp.112.196741. 22649272 PMC3425215

[B42] Lin B. L. Wang J. S. Liu H. C. Chen R. W. Meyer Y. Barakat A. (2001). Genomic analysis of the Hsp70 superfamily in *Arabidopsis thaliana*. *Cell Stress Chaperones* 6 201–208 10.1379/1466-1268(2001)006<0201:GAOTHS>2.0.CO;2 11599561 PMC434401

[B43] Lincoln J. E. Richael C. Overduin B. Smith K. Bostock R. Gilchrist D. G. (2002). Expression of the antiapoptotic baculovirus p35 gene in tomato blocks programmed cell death and provides broad-spectrum resistance to disease. *Proc. Natl. Acad. Sci. U.S.A.* 99 15217–15221 10.1073/pnas.232579799 12403830 PMC137570

[B44] Lisbona F. Rojas-Rivera D. Thielen P. Zamorano S. Todd D. Martinon F. (2009). BAX inhibitor-1 is a negative regulator of the ER stress sensor IRE1alpha. *Mol. Cell.* 33 679–691 10.1016/j.molcel.2009.02.017 19328063 PMC2818874

[B45] Liu J. X. Howell S. H. (2010a). bZIP28 and NF-Y transcription factors are activated by ER stress and assemble into a transcriptional complex to regulate stress response genes in *Arabidopsis*. *Plant Cell* 22 782–796 10.1105/tpc.109.072173 20207753 PMC2861475

[B46] Liu J. X. Howell S. H. (2010b). Endoplasmic reticulum protein quality control and its relationship to environmental stress responses in plants. *Plant Cell* 22 2930–2942 10.1105/tpc.110.078154 20876830 PMC2965551

[B47] Liu J. X. Srivastava R. Che P. Howell S. H. (2007a). An endoplasmic reticulum stress response in *Arabidopsis* is mediated by proteolytic processing and nuclear relocation of a membrane-associated transcription factor, bZIP28. *Plant Cell* 19 4111–4119 10.1105/tpc.106.050021 18156219 PMC2217655

[B48] Liu J. X. Srivastava R. Che P. Howell S. H. (2007b). Salt stress responses in *Arabidopsis* utilize a signal transduction pathway related to endoplasmic reticulum stress signaling. *Plant J.* 51 897–909 10.1111/j.1365-313X.2007.03195.x 17662035 PMC2156172

[B49] Liu J. X. Srivastava R. Che P. Howell S. H. (2008). Salt stress signaling in *Arabidopsis thaliana* involves a membrane-bound transcription factor AtbZIP17 as a signal transducer. *Plant Signal. Behav.* 3 56–57 10.4161/psb.3.1.4889 19704771 PMC2633961

[B50] Liu Y. Burgos J. S. Deng Y. Srivastava R. Howell S. H. Bassham D. C. (2012). Degradation of the endoplasmic reticulum by autophagy during endoplasmic reticulum stress in *Arabidopsis*. *Plant Cell* 24 4635–4651 10.1105/tpc.112.101535 23175745 PMC3531857

[B51] Liu Y. Schiff M. Czymmek K. Talloczy Z. Levine B. Dinesh-Kumar S. P. (2005). Autophagy regulates programmed cell death during the plant innate immune response. *Cell* 121 567–577 10.1016/j.cell.2005.03.007 15907470

[B52] Ma W. Berkowitz G. A. (2007). The grateful dead: calcium and cell death in plant innate immunity. *Cell Microbiol.* 9 2571–2585 10.1111/j.1462-5822.2007.01031.x 17714518

[B53] Ma W. Smigel A. Tsai Y. C. Braam J. Berkowitz G. A. (2008). Innate immunity signaling: cytosolic Ca2+ elevation is linked to downstream nitric oxide generation through the action of calmodulin or a calmodulin-like protein. *Plant Physiol.* 148 818–828 10.1104/pp.108.125104 18689446 PMC2556846

[B54] Maiuri M. C. Criollo A. Kroemer G. (2010). Crosstalk between apoptosis and autophagy within the Beclin 1 interactome. *EMBO J.* 29 515–516 10.1038/emboj.2009.377 20125189 PMC2830702

[B55] Martinon F. Glimcher L. H. (2011). Regulation of innate immunity by signaling pathways emerging from the endoplasmic reticulum. *Curr. Opin. Immunol.* 23 35–40 10.1016/j.coi.2010.10.016 21094031 PMC3042531

[B56] Mendes G. C. Reis P. A. Calil I. P. Carvalho H. H. Aragao F. J. Fontes E. P. (2013). GmNAC30 and GmNAC81 integrate the endoplasmic reticulum stress- and osmotic stress-induced cell death responses through a vacuolar processing enzyme. *Proc. Natl. Acad. Sci. U.S.A.* 110 19627–19632 10.1073/pnas.1311729110 24145438 PMC3845183

[B57] Nagashima Y. Mishiba K. Suzuki E. Shimada Y. Iwata Y. Koizumi N. (2011). *Arabidopsis* IRE1 catalyses unconventional splicing of bZIP60 mRNA to produce the active transcription factor. *Sci. Rep.* 1 29 10.1038/srep00029 PMC3216516 22355548

[B58] Nekrasov V. Li J. Batoux M. Roux M. Chu Z. H. Lacombe S. (2009). Control of the pattern-recognition receptor EFR by an ER protein complex in plant immunity. *EMBO J.* 28 3428–3438 10.1038/emboj.2009.262 19763086 PMC2776097

[B59] Noh S.-J. Kwon C. S. Chung W.-I. (2002). Characterization of two homologs of Ire1p, a kinase/endoribonuclease in yeast, in *Arabidopsis thaliana*. *Biochim. Biophys. Acta* 1575 130–134 10.1016/S0167-4781(02)00237-3 12020828

[B60] Oberstein A. Jeffrey P. D. Shi Y. (2007). Crystal structure of the Bcl-XL-Beclin 1 peptide complex: Beclin 1 is a novel BH3-only protein. *J. Biol. Chem.* 282 13123–13132 10.1074/jbc.M700492200 17337444

[B61] Qiang X. Zechmann B. Reitz M. U. Kogel K. H. Schafer P. (2012). The mutualistic fungus Piriformospora indica colonizes *Arabidopsis* roots by inducing an endoplasmic reticulum stress-triggered caspase-dependent cell death. *Plant Cell* 24 794–809 10.1105/tpc.111.093260 22337916 PMC3315247

[B62] Ranty B. Aldon D. Galaud J. P. (2006). Plant calmodulins and calmodulin-related proteins: multifaceted relays to decode calcium signals. *Plant Signal. Behav.* 1 96–104 10.4161/psb.1.3.2998 19521489 PMC2635005

[B63] Reed J. C. (1998). Bcl-2 family proteins. *Oncogene* 17 3225–3236 10.1038/sj.onc.1202591 9916985

[B64] Reis P. A. Fontes E. P. (2012). N-rich protein (NRP)-mediated cell death signaling: a new branch of the ER stress response with implications for plant biotechnology. *Plant Signal. Behav.* 7 628–632 10.4161/psb.20111 22580692 PMC3442856

[B65] Reis P. A. Rosado G. L. Silva L. A. Oliveira L. C. Oliveira L. B. Costa M. D. (2011). The binding protein BiP attenuates stress-induced cell death in soybean via modulation of the N-rich protein-mediated signaling pathway. *Plant Physiol.* 157 1853–1865 10.1104/pp.111.179697 22007022 PMC3327224

[B66] Rikiishi H. (2012). Novel insights into the interplay between apoptosis and autophagy. *Int. J. Cell Biol.* 2012 317645 10.1155/2012/317645 PMC3312193 22496691

[B67] Sano R. Reed J. C. (2013). ER stress-induced cell death mechanisms. *Biochim. Biophys. Acta* 1833 3460–3470 10.1016/j.bbamcr.2013.06.028 23850759 PMC3834229

[B68] Scorrano L. Oakes S. A. Opferman J. T. Cheng E. H. Sorcinelli M. D. Pozzan T. (2003). BAX and BAK regulation of endoplasmic reticulum Ca2+: a control point for apoptosis. *Science* 300 135–139 10.1126/science.1081208 12624178

[B69] Sinha S. Levine B. (2008). The autophagy effector Beclin 1: a novel BH3-only protein. *Oncogene* 27(Suppl. 1) S137–S148 10.1038/onc.2009.51 PMC2731580 19641499

[B70] Sparkes I. A. Frigerio L. Tolley N. Hawes C. (2009). The plant endoplasmic reticulum: a cell-wide web. *Biochem. J.* 423 145–155 10.1042/BJ20091113 19772494

[B71] Srivastava R. Chen Y. Deng Y. Brandizzi F. Howell S. H. (2012). Elements proximal to and within the transmembrane domain mediate the organelle-to-organelle movement of bZIP28 under ER stress conditions. *Plant J.* 70 1033–1042 10.1111/j.1365-313X.2012.04943.x 22335396

[B72] Srivastava R. Deng Y. Shah S. Rao A. G. Howell S. H. (2013). BINDING PROTEIN is a master regulator of the endoplasmic reticulum stress sensor/transducer bZIP28 in *Arabidopsis*. *Plant Cell* 25 1416–1429 10.1105/tpc.113.110684 23624714 PMC3663277

[B73] Stael S. Wurzinger B. Mair A. Mehlmer N. Vothknecht U. C. Teige M. (2012). Plant organellar calcium signalling: an emerging field. *J. Exp. Bot.* 63 1525–1542 10.1093/jxb/err394 22200666 PMC3966264

[B74] Sun L. Yang Z. T. Song Z. T. Wang M. J. Lu S. J. Liu J. X. (2013a). The plant-specific transcription factor gene NAC103 is induced by bZIP60 through a new cis-regulatory element to modulate the unfolded protein response in *Arabidopsis*. *Plant J.* 76 274–286 10.1111/tpj.12287 23869562

[B75] Sun Z. Yang D. Xie L. Sun L. Zhang S. Zhu Q. (2013b). Rice black-streaked dwarf virus P10 induces membranous structures at the ER and elicits the unfolded protein response in *Nicotiana benthamiana*. *Virology* 447 131–139 10.1016/j.virol.2013.09.001 24210107

[B76] Sung D. Y. Vierling E. Guy C. L. (2001). Comprehensive expression profile analysis of the *Arabidopsis* Hsp70 gene family. *Plant Physiol.* 126 789–800 10.1104/pp.126.2.789 11402207 PMC111169

[B77] Takeshige K. Baba M. Tsuboi S. Noda T. Ohsumi Y. (1992). Autophagy in yeast demonstrated with proteinase-deficient mutants and conditions for its induction. *J. Cell Biol.* 119 301–311 10.1083/jcb.119.2.301 1400575 PMC2289660

[B78] Urade R. (2009). The endoplasmic reticulum stress signaling pathways in plants. *Biofactors* 35 326–331 10.1002/biof.45 19415737

[B79] Valente M. A. Faria J. A. Soares-Ramos J. R. Reis P. A. Pinheiro G. L. Piovesan N. D. (2009). The ER luminal binding protein (BiP) mediates an increase in drought tolerance in soybean and delays drought-induced leaf senescence in soybean and tobacco. *J. Exp. Bot.* 60 533–546 10.1093/jxb/ern296 19052255 PMC2651463

[B80] Walter P. Ron D. (2011). The unfolded protein response: from stress pathway to homeostatic regulation. *Science* 334 1081–1086 10.1126/science.1209038 22116877

[B81] Watanabe N. Lam E. (2006). *Arabidopsis* Bax inhibitor-1 functions as an attenuator of biotic and abiotic types of cell death. *Plant J.* 45 884–894 10.1111/j.1365-313X.2006.02654.x 16507080

[B82] Watanabe N. Lam E. (2008). BAX inhibitor-1 modulates endoplasmic reticulum stress-mediated programmed cell death in *Arabidopsis*. *J. Biol. Chem.* 283 3200–3210 10.1074/jbc.M706659200 18039663

[B83] Williams B. Dickman M. (2008). Plant programmed cell death: can’t live with it; can’t live without it. *Mol. Plant Pathol.* 9 531–544 10.1111/j.1364-3703.2008.00473.x 18705866 PMC6640338

[B84] Williams B. Kabbage M. Britt R. Dickman M. B. (2010). AtBAG7, an *Arabidopsis* Bcl-2-associated athanogene, resides in the endoplasmic reticulum and is involved in the unfolded protein response. *Proc. Natl. Acad. Sci. U.S.A.* 107 6088–6093 10.1073/pnas.0912670107 20231441 PMC2851922

[B85] Williams B. Kabbage M. Kim H. J. Britt R. Dickman M. B. (2011). Tipping the balance: *Sclerotinia sclerotiorum* secreted oxalic acid suppresses host defenses by manipulating the host redox environment. *PLoS Pathog.* 7:e1002107. 10.1371/journal.ppat.1002107 PMC3128121 21738471

[B86] Xu H. D. Wu D. Gu J. H. Ge J. B. Wu J. C. Han R. (2013). The pro-survival role of autophagy depends on Bcl-2 under nutrition stress conditions. *PLoS ONE* 8:e63232. 10.1371/journal.pone.0063232 PMC3643928 23658815

[B87] Ye C. M. Chen S. Payton M. Dickman M. B. Verchot J. (2013). TGBp3 triggers the unfolded protein response and SKP1-dependent programmed cell death. *Mol. Plant Pathol.* 14 241–255 10.1111/mpp.12000 23458484 PMC6638746

[B88] Ye C. M. Kelly V. Payton M. Dickman M. B. Verchot J. (2012). SGT1 is induced by the potato virus X TGBp3 and enhances virus accumulation in *Nicotiana benthamiana*. *Mol. Plant* 5 1151–1153 10.1093/mp/sss026 22461666

[B89] Yorimitsu T. Nair U. Yang Z. Klionsky D. J. (2006). Endoplasmic reticulum stress triggers autophagy. *J. Biol. Chem.* 281 30299–30304 10.1074/jbc.M607007200 16901900 PMC1828866

[B90] Yoshida H. Matsui T. Yamamoto A. Okada T. Mori K. (2001). XBP1 mRNA is induced by ATF6 and spliced by IRE1 in response to ER stress to produce a highly active transcription factor. *Cell* 107 881–891 10.1016/S0092-8674(01)00611-0 11779464

[B91] Yue H. Nie S. Xing D. (2012). Over-expression of *Arabidopsis* Bax inhibitor-1 delays methyl jasmonate-induced leaf senescence by suppressing the activation of MAP kinase 6. *J. Exp. Bot.* 63 4463–4474 10.1093/jxb/ers122 22563118

[B92] Zambrano J. L. Ettayebi K. Maaty W. S. Faunce N. R. Bothner B. Hardy M. E. (2011). Rotavirus infection activates the UPR but modulates its activity. *Virol. J.* 8 359 10.1186/1743-422X-8-359 PMC3149005 21774819

[B93] Zhu X. Caplan J. Mamillapalli P. Czymmek K. Dinesh-Kumar S. P. (2010). Function of endoplasmic reticulum calcium ATPase in innate immunity-mediated programmed cell death. *EMBO J.* 29 1007–1018 10.1038/emboj.2009.402 20075858 PMC2837167

[B94] Zuppini A. Navazio L. Mariani P. (2004). Endoplasmic reticulum stress-induced programmed cell death in soybean cells. *J. Cell Sci.* 117 2591–2598 10.1242/jcs.01126 15159454

